# The NIHR Public Health Research Programme: responding to local authority research needs in the United Kingdom

**DOI:** 10.1186/s12961-015-0068-x

**Published:** 2015-12-11

**Authors:** Hannah Dorling, Andrew Cook, Liz Ollerhead, Matt Westmore

**Affiliations:** NIHR Evaluation Trials and Studies Coordinating Centre (NETSCC), University of Southampton, Alpha House, Enterprise Road, Southampton, SO16 7NS UK; Wessex Institute, University of Southampton, Southampton, UK; University Hospital Southampton NHS Foundation Trust, Southampton, UK

**Keywords:** Funding, Local government, Public Health, Research

## Abstract

The remit of the National Institute for Health Research Public Health Research (PHR) Programme is to evaluate public health interventions, providing new knowledge on the benefits, costs, acceptability and wider impacts of interventions, set outside of the National Health Service, intended to improve the health of the public and reduce inequalities. This paper illustrates how the PHR Programme is providing new knowledge for public health decision makers, based on the nine key areas for local authority public health action, described by the King’s Fund. Many funded PHR projects are evaluating interventions, applied in a range of settings, across the identified key areas for local authority influence. For example, research has been funded on children and young people, and for some of the wider determinants of health, such as housing and travel. Other factors, such as spatial planning, or open and green spaces and leisure, are less represented in the PHR Programme. Further opportunities in research include interventions to improve the health of adolescents, adults in workplaces, and communities. Building evidence for public health interventions at local authority level is important to prioritise and implement effective changes to improve population health.

## Background

The National Institute for Health Research (NIHR) is the research and development arm of the National Health Service (NHS). It is funded by the English Department of Health with contributions from the Chief Scientist Office in Scotland, Health and Care Research Wales, and the Health and Social Care Research and Development Public Health Agency in Northern Ireland. The NIHR aims to improve the health and wealth of the nation through research by providing a health research system in which the NHS supports outstanding individuals working in world-class facilities, conducting leading-edge research focused on the needs of patients and the public [1].

All NIHR funding programmes may fund research relevant to public health; however, most programmes are primarily focussed on healthcare settings, services and staff. Given the breadth of public health, a gap was identified in the funding of high quality, nationally important evaluations of interventions set outside of the NHS [2]. The NIHR Public Health Research (PHR) Programme was established to fill this gap in 2008. The remit of the PHR Programme is to fund evaluations on public health interventions, providing new knowledge on the benefits, costs, acceptability and wider impacts of non-NHS interventions intended to improve the health of the public and reduce inequalities in health [3]. The scope of the Programme is multi-disciplinary and broad, covering a wide range of public health interventions. The Programme is funded by the governments of all four United Kingdom countries.

The Programme aims to identify gaps in knowledge and fund research that provides high-quality evidence to fill such gaps [4]. PHR funds research through two routes – commissioned, where the Programme highlights an area that has the potential for developing new evidence, and researcher-led, where a research question is proposed by researchers. The Programme employs a needs-led approach to ensure that research is of the highest possible value to public health decision-makers, such as those working in local authorities. By this, we mean that the Programme prioritises funding research according to the needs of service providers and policymakers whose decisions can impact on the public’s health. For example, the Programme Advisory Board, which is comprised of public health decision-makers, prioritises topics for future research and assesses submitted research applications for their potential importance in improving the evidence base for decision-making.

From its inception to June 2014, the PHR Programme had funded 83 projects. The portfolio of studies, including protocols and final reports, can be found at: www.nets.nihr.ac.uk/projects/phr. Although 5 years was a relatively short period for a funding programme, there were a sufficient number of studies to start reflecting on the balance of the portfolio and its future direction. This paper aims to describe the breadth of the portfolio, particularly in funded research which might inform local authorities. We use a framework for our description – the nine areas for local authority action identified in a recent report by the King’s Fund [5]. The nine key areas that can improve public health and reduce inequalities are ‘The best start in life’, ‘Healthy schools and pupils’, ‘Helping people find good jobs and stay in work’, ‘Active and safe travel’, ‘Warmer and safer homes’, ‘Access to green and open spaces and the role of leisure services’, ‘Strong communities, wellbeing and resilience’, ‘Public protection and regulatory services’, and ‘Health and spatial planning’ (Table 1). The King’s Fund is a leading independent health think tank in England, which is set up as a charity working to improve health and healthcare. Their vision is that the best possible healthcare is available to all [6], which involves providing advice or ideas to organisations to bring about change. There is no formal relationship between the King’s Fund and the NIHR, but it is important for research funders, such as the NIHR, to be aware of the work the King’s Fund is undertaking, as it may indicate the evidence user needs.

**Table 1:** Projects categorised by the nine areas for local authority action identified by the King’s Fund

Project area	Total number of projects, n (%)	Commissioned, n	Researcher-led, n
The best start in life	9 (11)	5	4
Healthy schools and pupils	22 (27)	11	11
Helping people find jobs and stay in work	2 (2)	2	0
Active and safe transport	7 (8)	3	4
Warmer and safer homes	5 (6)	1	4
Access to green and open spaces and the role of leisure services	5 (6)	2	3
Strong communities, wellbeing and resilience	4 (5)	4	0
Public protection and regulatory services	7 (8)	1	6
Health and spatial planning	3 (4)	0	3
Other	19 (23)	9	10
Total	83	38	45

## Review

The table below displays the spread of projects across the nine key areas, showing whether the project was funded through the commissioned or researcher-led work-stream.

Most projects are centred on the theme of ‘Healthy schools and pupils’, closely followed by ‘The best start in life’, ‘Active and safe transport’ and ‘Public protection and regulatory services’. The other categories all have funded projects, but still provide an opportunity to fill important gaps in research. The ‘Other’ category represents projects which do not fall within the nine action areas. Local authorities are one of the main customers of the PHR Programme, but there are other customers, such as the third sector and health promotion services.

The commissioned work-stream represents 46% of the funded projects and the researcher-led work-stream represents 54% of the funded projects. Higher numbers of projects, from the commissioned work-stream, in particular areas may suggest topics with research interest or capacity. However, some research topics are particularly suited to the researcher-led work-stream, as opportunities arise. For example, changes to the built environment, shown in the ‘Health and spatial planning’ category, which are often evaluated as natural experiments. The commissioned work-stream encourages research for specific topics and populations to create a portfolio that fills important gaps in evidence.

### The best start in life

There are nine research projects on early years interventions that feature in the PHR portfolio. The King’s Fund recommends possible priority actions throughout their report [5]. For the best start in life, it is suggested that the most disadvantaged children and their families are targeted for support and interventions should be behaviour-focused, rather than just information-giving. Following the identification of an evidence gap and advertisement for proposals, PHR have funded a range of projects which involve parenting support programmes, with a particular focus on vulnerable children and families. This includes projects assessing the Family Nurse Partnership [7, 8] and improving outcomes for children who are exposed to domestic abuse or maltreatment [9, 10].

Enabling the best start in life is also addressed by support for positive health behaviours. ‘Pre-schoolers in the Playground’ is a pilot study of an intervention which aims to increase physical activity levels in children aged 18 months to 4 years old, funded through a commissioned call on outdoor community activity programmes. The intervention includes opening up school playgrounds for pre-school siblings to use as a safe place to take part in outdoor physical activity, whilst fitting in with the family’s school run [11]. Another funded study to try to increase physical activity and improve nutrition in early years is the Nutrition and Physical Activity Self Assessment for Child Care. This programme trains staff who work in early years’ settings to assess and improve the child care environment with respect to food, drink and physical activity [12].

### Healthy schools and pupils

The King’s Fund recommends promoting the school as a setting for healthy behaviours. Twenty-two PHR projects evaluate interventions delivered in schools. These include programmes to increase levels of physical activity, decrease sedentary behaviour, improve diet, prevent hazardous drinking, smoking and substance use, promote sexual health, build social and emotional wellbeing, and reduce bullying. Schools can be a valuable setting for public health interventions, as there are possibilities to intervene during lesson time or through extra-curricular activities, and to include families and communities. They have also been acknowledged as potentially effective settings for public health interventions, as they reach a large number of children and adolescents [13] and during the school year children spend a significant number of their waking hours at school [14]. This establishes an opportunity to promote health and wellbeing for all children, irrespective of their circumstances, developing knowledge and skills for the foundations for future health.

One of the areas the programme has concentrated on is reducing alcohol harm. The NICE Public Health Guidance for school-based interventions on alcohol noted a gap in evidence on the effectiveness of interventions for children and young people [15]. As this research question was viewed as an important gap to address by NICE, and in the remit of the PHR Programme, a commissioning brief was developed and advertised on interventions to prevent hazardous drinking of alcohol by school-aged children and young people. Three studies were awarded funding to address specific research questions in this area, using school-based interventions but involving families [16–18]. However, there are many other areas which can potentially influence positive behaviours for young people at school, such as changing the school environment to improve health behaviours. Although interventions in schools are well represented in the PHR portfolio, there are still many opportunities to extend the range of health topics. For example, further and higher education institutions may be a key educational setting to help young people improve their health; however, there is currently only one project in the PHR portfolio that uses a further education setting.

### Helping people find good jobs and stay in work

The workplace provides an opportunity for improving health, as there is potential to reach large audiences, and having good working conditions is well known as a determinant of health [19]. Workplaces in the United Kingdom are moving towards a more active approach to reducing sickness absence. For instance, both the Black review of the health of the working age population [20] and the Black and Frost Independent Review of Sickness Absence [21], highlight the benefit of health and wellbeing programmes for businesses economically, and the importance of reducing sickness absence. The King’s Fund recommends a number of actions to improve the health of employees such as promoting health-enhancing work cultures and implementing effective health promotion initiatives. The Programme has funded research projects in the workplace, including interventions aimed at encouraging walking to work [22] and reducing sickness absence [23]. There are many opportunities for further public health research in this area, such as promoting work cultures and environments to support the health of employees. A systematic review that recently explored workplace health promotion interventions for increasing physical activity showed some evidence that workplace physical activity interventions can be effective, although the overall results were inconclusive and there is a need for more robust studies [24]. Workplace health remains a priority area for research in the PHR Programme.

### Active and safe travel

Transport can affect health positively by increasing physical activity levels and reducing isolation, or negatively, through transport injuries and air pollution. A range of studies have been funded by PHR on the public health impacts of infrastructure and provision, such as the effects of a new urban motorway [25], the health impacts of the Cambridgeshire Guided Busway [26] and ‘On the buses’ [27], which evaluated the impact of introducing free bus travel for young people with benefits for health and social inclusion. Other studies evaluate interventions which may affect safety, such as those aimed at maximising cycling safety [28], and assessing how reducing street lighting may affect crime and road traffic accidents at night [29]. The King’s Fund report gives a number of recommendations, including changing public perception about cycling safety, cycle to work schemes, 20 mile per hour zones and changing the environment to improve walkability. These interventions could all be potentially researched to build a picture of the effectiveness and cost to local authorities. Research on transport remains a high priority for future health and sustainability, and further evidence will be important for local authorities to continue developing active and safe travel.

### Warmer and safer homes

The King’s Fund priority action areas include helping people keep their homes warmer and preventing accidents in the home. Several projects have been funded in this area, including the health impacts of structural energy performance investments in Wales [30], the impact of home energy efficiency interventions and winter fuel payments on winter- and cold-related mortality and morbidity in England [31], the health impact of meeting housing quality standards [32], and manipulating the appearance of steps and stairs to make them safer for older people to negotiate [33]. To build evidence relating to the King’s Fund recommendations, further research is needed on how to prevent unintentional injuries in the home and how to provide healthy housing across the life-course, and for people with particular needs.

### Access to green and open spaces and the role of leisure services

Access to green, open spaces and leisure services can shape people’s physical and mental health, and build networks. One study from the PHR portfolio is evaluating whether changes to the natural environment can help psychological wellbeing [34]. Another project which uses green spaces includes understanding the impacts of care farms on health and wellbeing of offenders [35]. This aims to evaluate farming activities to help build self-esteem, improve physical and mental health, develop skills for employment and increase ability to interact socially.

The importance of creating more good quality open space, where it is lacking, has also been discussed in the Marmot Review as a means of tackling health inequalities [36]. However, the evidence in this area is not strong [37], and there are many opportunities for further research to help local authorities. Interventions designed to increase access to green and open spaces requires further evaluation; for instance, the King’s Fund recommends working with local communities to help develop plans to help stimulate physical activity levels and engaging with community groups in the management of green spaces.

### Strong communities, wellbeing and resilience

Community based interventions feature heavily in the PHR portfolio of funded projects. This approach aims to address some of the causes of inequalities, and can empower a large number of people. For instance, two research projects evaluate community engagement as an important way of developing public services that better meet people’s needs and increase social cohesion [38, 39]. Other research evaluates interventions for specific communities [40], such as outreach programmes for health improvement of traveller communities. The research explores interventions that are adapted and taken to populations who do not, or cannot, access them as they are traditionally provided [39]. Potentially, there are possibilities for research, with other communities and with a different range of interventions. The King’s Fund suggests building social capital and community-based assets to improve health, for example, by supporting volunteering interventions and working with wider public services. This may benefit from further research on the most effective ways of types of interventions and their implementation in communities.

### Public protection and regulatory services

Protective and regulatory services can be key components to support health improvement. The King’s Fund report [5] focuses on the regulation of takeaways and fast foods, the improvement of air quality and fire safety. Another area of public protection that the programme has funded is research that investigates the impact of a change in the density of alcohol outlets on alcohol consumption and alcohol-related harms to health in the community [41]. The findings from this study may potentially help local authorities consider changes to alcohol outlet density in the community to help improve health and reduce crime. Air pollution is a risk factor for a number of health problems, particularly for those with existing health conditions, or those who live in areas with poorer air quality. This is a complex area for assessing interventions. However, there is currently a project looking at the impacts of different pathways to meet the United Kingdom Climate Change Act commitment to 80% reduction on CO_2_ and other greenhouse gas emissions by 2050 [42]. Further research on the areas suggested by the King’s Fund, and on other approaches for protective and regulatory services to improve population health and reduce health inequalities, could be helpful to local authorities.

### Health and spatial planning

There is growing interest on the impact of the built environment on health [43]. This interest is reflected in the PHR portfolio, where the results can influence local authorities’ decisions on spatial planning. All of the funded studies in this area have been defined by researchers, from the researcher-led work-stream. They cover a wide range of interventions, such as active design to increase physical activity levels [44] and the impact of urban regeneration on the social determinants of health, health behaviours and mental health [45].

A PHR funded research project, which has recently been published, conducted a systematic review to synthesize qualitative evidence from the United Kingdom on fear of crime and the environment [46]. The review suggested that any attempt to address fear of crime through the physical environment in isolation is unlikely to succeed, and consideration would need to be given to the social environment, or the socioeconomic or policy context, to establish positive changes. The findings of this study may be useful to help inform decision makers. However, there are still many opportunities to evaluate interventions in this broad area such as indoor air quality, building conditions and infrastructure design for health.

### Reflection on the portfolio of research

This analysis provides a snapshot of the portfolio of funded research. The King’s Fund nine key action areas were chosen as a framework as it is recent and relevant to the main customers of the PHR Programme. The King’s Fund has noted the strengths of interventions in each area that allows reflection on which interventions will deliver the best results [5]. Over a quarter of the projects fall within the ‘Healthy Schools and Pupils’ category, although there still remain gaps within this category. For example, the consideration of different settings within education such as further and higher education. Other categories which follow closely behind include ‘The best start in life’, ‘Active and safe transport’ and ‘Public protection and regulatory services’. The King’s Fund report highlights that these interventions may have a significant impact to improve health and reduce inequalities, but they require more investment and it may take a long time to demonstrate impact.

The categories with the least amount of projects include ‘Helping people find jobs’ and ‘Health and spatial planning’. However, the report highlights that all categories are strong candidates for action to improve population health and shows how health determinants may affect one another. As a result, these may need further analysis to consider the most important gaps to fill within these areas for local authorities, using the needs-led prioritisation process. Possible topics for research are reviewed by advisory groups of external experts and public members to assess the need for the research.

It is important to consider the impact of the research. However, as much of the research has not yet been completed or has only recently been published, this will be assessed at a later time. Interest in the research findings can be assessed now by process and surrogate measures, such as recording the number of report downloads, citations, collecting case studies and using online platforms, such as Researchfish (www.researchfish.com), which enables research funders to track the impacts of their investments. An external review may be commissioned, such as the Raftery, Hanney and Buxton paper, which assesses the impact of the NHS Health Technology Assessment Programme in England [47].

## Conclusions

Building evidence for public health interventions at local authority level is important to prioritise and implement effective changes to improve population health. This paper provides an opportunity to reflect on the diversity of public health interventions outside of the NHS in the PHR Programme, based on the nine key areas for local authority public health action, as described by the King’s Fund. The programme has an expanding portfolio of relevant and potentially useful research for local authorities. The ‘Best start in life’, ‘Healthy schools and pupils’, ‘Active and safe travel’, and ‘Warmer and safer homes’ appear to be better represented than other key areas. When reflecting on the potential impact that the interventions could have, all are in categories which the King’s Fund report suggests are likely to be highly important for their impact on population health. There are many opportunities for researchers to fill gaps in evidence for local authorities by providing new knowledge and give further breadth to the PHR portfolio.

The authors of this review invite researchers to submit proposals relating to the evidence gaps identified within the remit of the PHR Programme, or other NIHR programmes, by submitting an application to the researcher-led work-stream. The Programme would also like to encourage submission of suggestions for potential future research www.nets.nihr.ac.uk/identifying-research/make-a-suggestion.

## Abbreviations

PHR: Public Health Research
NHS: National Health Service
NIHR: National Institute for Health Research
